# Evaluation of Brain Alterations and Behavior in Children With Low Levels of Prenatal Alcohol Exposure

**DOI:** 10.1001/jamanetworkopen.2022.5972

**Published:** 2022-04-05

**Authors:** Xiangyu Long, Catherine Lebel

**Affiliations:** 1 Department of Radiology, University of Calgary, Calgary, Alberta, Canada; 2 Alberta Children’s Hospital Research Institute, Calgary, Alberta, Canada; 3 Hotchkiss Brain Institute, University of Calgary, Calgary, Alberta, Canada

## Abstract

**Importance:**

High levels of prenatal alcohol exposure (PAE) are associated with widespread behavioral and cognitive problems as well as structural alterations of the brain. However, it remains unclear whether low levels of PAE affect brain structure and function, and prior studies generally have not had well-matched control populations (eg, for sociodemographic variables).

**Objective:**

To compare structural brain alterations and behavioral changes in children with lower levels of PAE with those of well-matched controls with no PAE.

**Design, Setting, and Participants:**

In this cross-sectional study, participants were selected from the Adolescent Brain Cognitive Development study. Children with PAE were compared with controls matched for age, sex, family income, maternal educational level, and caregiver status. Neither group had prenatal exposure to other adverse substances (eg, tobacco, cannabis, illicit drugs). Data were collected from September 1, 2016, to November 15, 2018, and analyzed from October 14, 2020, to February 14, 2022.

**Exposures:**

Diffusion tensor imaging, resting-state functional magnetic resonance imaging (MRI), and Child Behavior Checklist (CBCL) administration.

**Main Outcomes and Measures:**

Fractional anisotropy (FA); mean, axial, and radial diffusivity from diffusion tensor imaging; brain functional signal variations from functional MRI; and several scores, including internalizing and externalizing behavior problems, from the CBCL. Spearman correlation coefficients between diffusion tensor imaging and functional MRI measures and the CBCL scores were calculated.

**Results:**

A total of 270 children were included in the analysis (mean [SD] age, 9.86 [0.46] years; 141 female [52.2%] and 129 male [47.8%]), consisting of 135 children with PAE (mean [SD] age, 9.85 [0.65] years; 73 female [54.1%] and 62 male [45.9%]) (mean exposure, 1 drink/wk) and 135 unexposed controls (mean [SD] age, 9.87 [0.04] years; 68 female [50.4%] and 67 male [49.6%]). Children with PAE had lower mean (SD) FA in white matter of the left postcentral (0.35 [0.05] vs 0.36 [0.04]; mean difference, −0.02 [95% CI, −0.03 to −0.01]), left inferior parietal (0.31 [0.07] vs 0.33 [0.06]; mean difference, −0.03 [95% CI, −0.04 to −0.01]), left planum temporale (0.26 [0.04] vs 0.28 [0.03]; mean difference, −0.02 [95% CI, −0.03 to −0.01]), left inferior occipital (0.30 [0.07] vs 0.32 [0.05]; mean difference, −0.03 [95% CI, −0.04 to −0.01]), and right middle occipital (0.30 [0.04] vs 0.31 [0.04]; mean difference, −0.01 [95% CI, −0.02 to −0.01]) areas compared with controls, and higher FA in the gray matter of the putamen (0.22 [0.03] vs 0.21 [0.02]; mean difference, 0.01 [95% CI, 0.005-0.02]). Externalizing behavior scores were higher (worse) in children with PAE than in controls (mean [SD], 45.2 [9.0] vs 42.8 [9.0]; mean difference, 2.39 [95% CI, 0.30-4.47]). Several of these regions had significant group-behavior interactions, such that the higher FA was associated with less problematic behaviors in controls (ρ range, −0.24 to −0.08) but no associations were present in the PAE group (ρ range, 0.02-0.16).

**Conclusions and Relevance:**

In this cross-sectional study, children with low levels of PAE had lower FA and more behavioral problems compared with a well-matched control group. These results suggest that PAE, even in small amounts, has a measurable effect on brain structure in children.

## Key Points

**Question:**

How do brain structure and behavior of children with low levels of prenatal alcohol exposure (PAE) differ compared with those of a well-matched control group of children?

**Findings:**

In this cross-sectional study of 135 children with low levels of PAE and 135 unexposed controls, children with PAE had worse externalizing behavior scores and lower fractional anisotropy in several white matter areas of the brain compared with children in the control group. Higher fractional anisotropy was associated with less problematic behavior in the control group, but no association was found in the PAE group.

**Meaning:**

These findings suggest that even small amounts of PAE are associated with structural brain alterations, highlighting the importance of evidence-based policy making and underscoring the need to consider prenatal exposures in future studies of pediatric brain development.

## Teaser

This cross-sectional study examines altered brain structure and function in children with low levels of prenatal alcohol exposure compared with well-matched unexposed children.

## Introduction

Nearly 10% of pregnant individuals report consuming alcohol,^1^ which can adversely affect fetal development and lead to a variety of physical, behavioral, and neurological deficits.^2^ Heavy prenatal alcohol exposure (PAE) is associated with a diagnosis of fetal alcohol spectrum disorder (FASD), a neurodevelopmental disorder that includes lifelong cognitive and behavioral difficulties.^2,3^ Prenatal alcohol exposure is also is associated with an increased risk of mental health problems; more than 90% of individuals with FASD have a co-occurring mental illness.^4^

Using magnetic resonance imaging (MRI), disrupted brain structure and function after PAE have been observed. The most common finding is reduced brain volume, which has been observed among individuals ranging from infants to adults.^5,6^ Altered white matter microstructure has also been reported across ages using diffusion tensor imaging, although neonates and young children with PAE tend to show lower diffusivity,^7,8^ whereas older children, adolescents, and young adults with PAE show higher diffusivity and lower anisotropy^9,10,11^ and weaker structural network connectivity^12^ than controls. In terms of brain function, increased functional connectivity among motor, brainstem, and thalamic networks have been detected in neonates with PAE.^13,14^ In children and adolescents, PAE is associated with disrupted functional connectivity in the default mode network,^15^ sensorimotor network,^16^ attention and language network,^17,18,19,20^ and whole brain network structure.^21,22^

Internalizing behaviors (those directed inward such as depression and anxiety) and externalizing behaviors (those directed outward such as aggression and hyperactivity) are more common in children with PAE and can indicate a higher risk of later mental illness.^4,23^ In unexposed children and adolescents, these behaviors are related to white matter microstructure,^24^ cortical thickness,^25^ and gray matter volumes.^26,27^ However, few studies have examined associations between adverse behaviors and brain alterations associated with PAE. One study with a relatively small sample^28^ showed no association between brain volumes or white matter microstructure and internalizing or externalizing behavior, whereas another study^29^ found negative correlations between brain volumes and psychopathology and behavioral scores in youths with PAE.

Most previous studies of brain alterations and PAE have focused on individuals with high levels of PAE and/or a diagnosis of FASD (which requires high levels of exposure or the sentinel facial features associated with fetal alcohol syndrome). However, lower levels of PAE can affect cognitive and behavioral function,^30,31^ and emerging evidence^29,32^ suggests brain volumes and functional connectivity are affected. Dose-dependent associations between the amount of PAE and brain volumes have been reported by several prior studies, providing further evidence that lower levels of exposure may impact brain volumes.^33,34,35^ White matter tends to be disproportionately affected by PAE,^5,36^ yet it remains unclear how low levels of PAE are associated with white matter microstructure and structural brain connectivity. The lack of evidence of structural changes in the brain associated with low levels of PAE may be part of the reason that many individuals perceive small amounts of PAE as safe during pregnancy. Understanding how low levels of PAE affect brain outcomes is critical for making evidence-based policy recommendations.

Most prior studies of PAE^28,34,37,38,39,40^ have been unable to appropriately control for factors such as family income, educational level, child care situation, and prenatal exposure to other substances owing to small sample sizes. For example, in many studies,^28,34,37^ most children with PAE and/or FASD are in adoptive or foster care, whereas the control group primarily resides with biological parents. Many studies^38,39,40^ also report lower family income and/or parent educational level in the PAE group compared with controls. Prenatal exposure to other teratogenic substances such as tobacco, cannabis, and illicit drugs often co-occurs with PAE,^41^ and these substances are independently associated with brain alterations.^42,43,44^ Studies with well-matched controls and alcohol exposure in isolation are critical to better understand the specific effects of PAE.

The aim of the present study was to examine brain structure and function in children with low to average levels of PAE while carefully controlling for potentially confounding factors. We hypothesized that children with PAE would have lower total brain volume, lower fractional anisotropy (FA), and higher functional MRI signal variations compared with unexposed matched controls. Furthermore, we expected correlations between brain metrics and internalizing and externalizing behavioral scores, such that lower FA would be associated with worse behavior.

## Methods

### Participants

All neuroimaging and cognitive and behavioral data were obtained from the Adolescent Brain Cognitive Development (ABCD) study, release 2.0.1.^45^ This cohort study recruited more than 10 000 children approximately 10 years of age across the US from September 1, 2016, to November 15, 2018. Neuroimaging data include structural MRI, resting-state functional MRI, and diffusion tensor imaging. The ABCD study was approved by a central institutional review board at the University of California, San Diego, with local institutional review board approval for a few sites.^46^ Parents provided written informed consent and children provided informed assent. The present study followed the Strengthening the Reporting of Observational Studies in Epidemiology (STROBE) reporting guideline.

The ABCD Developmental History Questionnaire asked about PAE and prenatal exposure to other substances (tobacco, cannabis, heroin, cocaine, and oxycodone [Oxycontin]).^47^ Using these data, we identified participants whose biological mothers (adoptive parents were excluded owing to potential lack of knowledge of prenatal exposures) answered yes to using alcohol and no to using other substances (tobacco, cannabis, heroin, cocaine, and oxycodone) after awareness of their pregnancy (n = 135). Children whose biological mothers answered no to using alcohol and other substances both before and after awareness of their pregnancy were selected as control participants. Controls were matched to the exposure group on sex, age, maternal educational level, family income, biological parents, and no prenatal exposure to other substances (Table 1). Family income for the past 12 months was measured on a 10-point scale from less than $5000 to greater than $200 000. Maternal educational level was reported on a 21-point scale ranging from never attended school to a doctoral degree.

**Table 1.** Demographics of the Participants in the Present Study

Characteristic	PAE group (n = 135)	Matched unexposed controls (n = 135)^a^	*P* value, difference between groups	Cohen *d*
Age, mean (SD), y	9.85 (0.65)	9.87 (0.04)	.73	0.04
Sex, No. female/male	73/62	68/67	.94	0.07
No. of study sites	22	21	.42	0.01
No. of scanner types	3	3	.94	0.39
Total family income, mean (SD)^b^	9 (2)	9 (0)	.18	0.16
Maternal educational level, mean (SD)^c^	18 (2)	18 (0)	.50	0.08
Maximum No. of drinks in 1 sitting, mean (SD)	1 (1)	NA	NA	NA
No. of drinks per week, mean (SD)	1 (1)	NA	NA	NA

Abbreviations: NA, not applicable; PAE, prenatal alcohol exposure.

^a^ The unexposed controls were selected to match the PAE group for age, sex, family income, and maternal educational level. Neither group had prenatal exposure to any other adverse substances (eg, tobacco, cannabis, illicit drugs).

^b^ Measured on a 10-point scale from less than $5000 to greater than $200 000, with higher scores indicating higher income.

^c^ Measured on a 21-point scale ranging from never attended school to a doctoral degree, with higher scores indicating higher educational level.

To assess the quantity of prenatal exposure, we used the answers to: “average drinks per week?” and “maximum drinks in 1 sitting?” after knowing of pregnancy (Table 1). One hundred fourteen of the 135 participants with PAE had information available on drinking quantities.

### Behavior Measures

The Child Behavior Checklist (CBCL) was used to assess internalizing and externalizing behaviors.^47^ The summary internalizing, externalizing, and total problem scores, as well as scores of 8 individual scales (Anxious/Depressed, Withdrawn/Depressed, Somatic Complaints, Social Problems, Thought Problems, Attention Problems, Rule-Breaking Behavior, and Aggressive Behavior) were compared between groups. For all participants, behavior measures were collected on the same day as the neuroimaging session.^47^

### Neuroimaging

In brief, diffusion tensor imaging preprocessing included corrections for eddy current distortions and head motion. The functional MRI images were preprocessed with head motion correction, normalization, nuisance signal removal (ie, from white matter, ventricles, and the global signal), and band-pass filtering of 0.009 to 0.08 Hz. We corrected T1-weighted images for gradient nonlinearity distortions and intensity inhomogeneity. Cortical surface reconstruction on the T1-weighted images was performed by FreeSurfer brain imaging software, version 5.3 (Harvard University); this was used to calculate total brain volume and create cortical surface-based regions of interest.^45,48^ The ASEG atlas from FreeSurfer was used to segment deep gray matter regions.^49^ A more detailed description of ABCD study data processing methods is available elsewhere.^48^

Our analysis used the tabulated MRI measurements of the first time point released from the ABCD study for each participant. Mean FA, mean diffusivity, axial diffusivity, and radial diffusivity were calculated across each of the subadjacent white matter areas of cortical regions from FreeSurfer,^50^ as well as the subcortical gray matter regions. The mean temporal variance of functional MRI blood oxygenation level–dependent signal was calculated within each cortical gray matter Destrieux region.^50,51^ We also extracted total brain volume.

### Statistical Analysis

Data were analyzed from October 14, 2020, to February 14, 2022. The present study used 2-sample *t* tests, linear mixed models, and Spearman correlation analysis. Results that were both corrected and uncorrected for false discovery rate at a significance level of 2-sided *P* < .05 are reported along with mean differences and 95% CIs. Group comparisons between the participants with PAE and unexposed controls were performed on the CBCL scores and MRI measurements of each brain region using a series of 2-sample *t* tests. Site was included as a random effect for MRI measurements.^52^

Brain regions with significant group differences in MRI metrics were then examined for associations with CBCL behavior scores. Spearman correlation analysis was performed between MRI metrics and CBCL scores when controlling site as a random effect, then the correlation coefficients were transformed to *z* scores and compared between groups.

## Results

### Alcohol Consumption

A total of 270 children (mean [SD] age, 9.86 [0.46] years; 141 female [52.2%] and 129 male [47.8%]) were included in the analysis; 135 children (mean [SD] age, 9.85 [0.65] years; 73 female [54.1%] and 62 male [45.9%]) with PAE were compared with 135 controls (mean [SD] age, 9.87 [0.04] years; 68 female [50.4%] and 67 male [49.6%]). Mean (SD) PAE in the sample was 1 (1) drink/wk. The mean (SD) number of drinks in a single sitting was 1 (1). Most participants’ biological mothers (112 [98.3%]) reported no binge episodes (defined as ≥4 drinks in 1 sitting) during pregnancy. Controls reported no alcohol exposure at any time during pregnancy (before or after pregnancy recognition).

### CBCL Scores Related to Prenatal Exposure

Most participants in both the PAE and unexposed control groups scored in the normal range of behaviors (ie, scores <65). The PAE group had significantly elevated (ie, worse) externalizing behavior scores than controls (mean [SD], 45.2 [9.0] vs 42.8 [9.0] ]; mean difference, 2.39 [95% CI, 0.30-4.47]); 2 participants with PAE (1.5%) had clinically elevated externalizing scores (≥65), whereas no controls had clinically elevated scores (eTable 1 in the Supplement). There were no other significant differences in internalizing scores or CBCL subscale scores between groups.

### Neuroimaging Metrics Associated With PAE

The PAE group had significantly higher mean (SD) total cortical brain volume than unexposed controls (624 [64] vs 605 [55] cm^3^; mean difference, 18.77 [95% CI, 4.55-33.00] cm^3^; *P* = .01), but no difference in mean (SD) total intracranial volume (1557 [174] vs 1544 [134] cm^3^; mean difference, 14.15 [95% CI, −23.24 to 51.55] cm^3^; *P* = .46).

**Figure 1.**
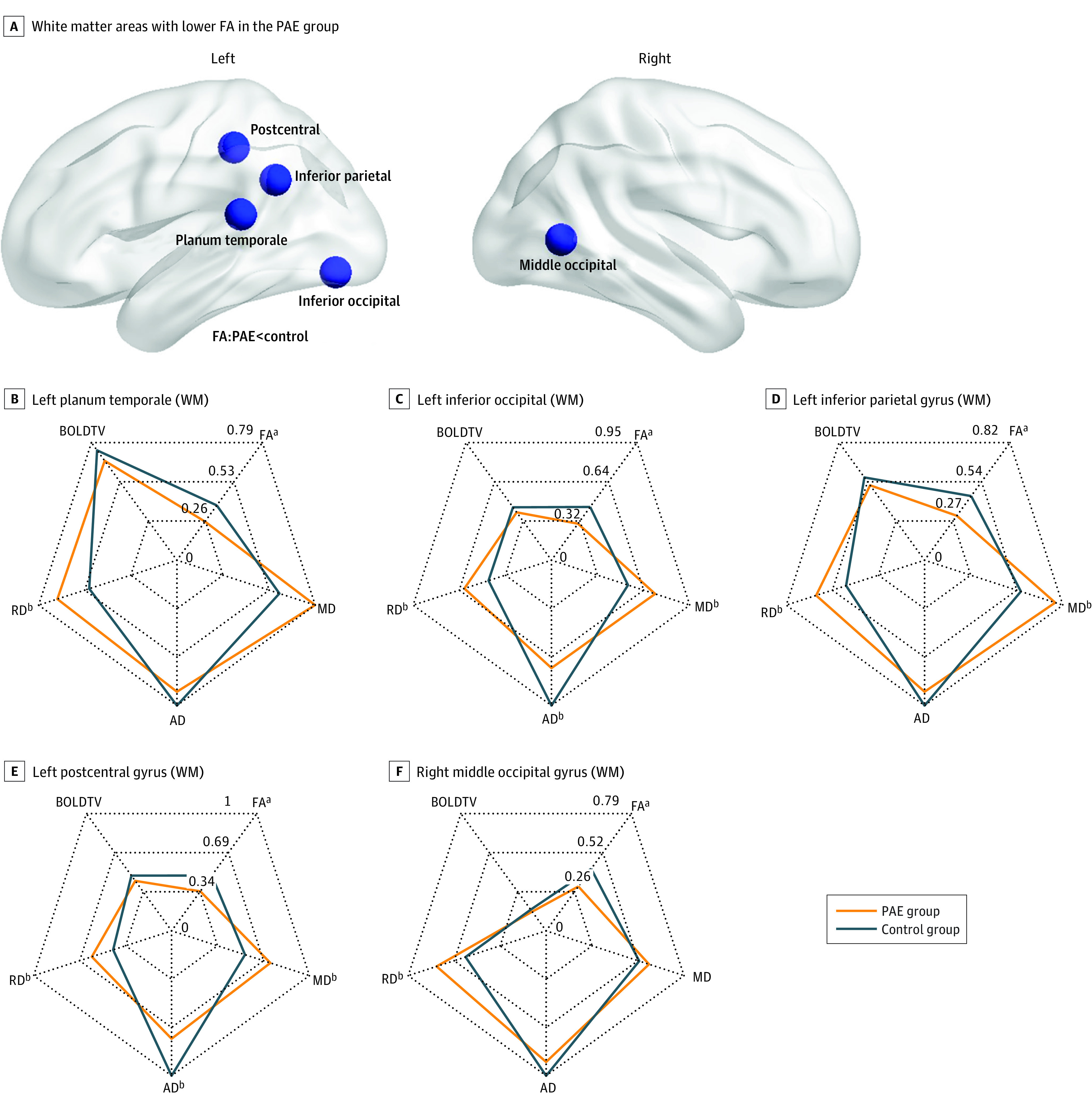
White Matter Areas With Lower Fractional Anisotropy (FA) in the Prenatal Alcohol Exposure (PAE) Group Compared With Unexposed Controls A, White matter areas with lower FA in the PAE group compared with unexposed controls. B-F, Magnetic resonance imaging metrics (rescaled) of selected white matter (WM) regions by group. AD indicates axial diffusivity; BOLDTV, blood oxygenation level–dependent signal temporal variance; MD, mean diffusivity; and RD, radial diffusivity. ^a^Group differences corrected for false discovery rate at *P* < .05. ^b^Group differences uncorrected for false discovery rate at *P* < .05.

The mean (SD) FA in the following 5 white matter regions was significantly lower in the PAE group compared with matched controls after false discovery rate correction (Table 2 and Figure 1): the left postcentral (0.35 [0.05] vs 0.36 [0.04]; mean difference, −0.02 [95% CI, −0.03 to −0.01]), left inferior parietal (0.31 [0.07] vs 0.33 [0.06]; mean difference, −0.03 [95% CI, −0.04 to −0.01]), left planum temporale (0.26 [0.04] vs 0.28 [0.03]; mean difference, −0.02 [95% CI, −0.03 to −0.01]), left inferior occipital (0.30 [0.07] vs 0.32 [0.05]; mean difference, −0.03 [95% CI, −0.04 to −0.01]), and right middle occipital (0.30 [0.04] vs 0.31 [0.04]; mean difference, −0.012 [95% CI, −0.02 to −0.01]) areas. Higher FA in the gray matter of the left putamen was also found in children with PAE (0.22 [0.03] vs 0.21 [0.02]; mean difference, 0.01 [95% CI, 0.005-0.02]). These white matter regions also had higher radial diffusivity, higher mean diffusivity, and/or lower axial diffusivity in the PAE group, although these findings did not survive multiple comparison correction (Figure 1B-F).

**Table 2.** Brain Areas With Significant FA Differences Between Groups After Correction for False Discovery Rate^a^

Brain hemisphere	Brain region	FA, mean (SD)		Mean difference (95% CI)	*P* value	Cohen *d*
		PAE group	Matched unexposed controls			
Left	Planum temporale (white matter)	0.26 (0.04)	0.28 (0.03)	−0.02 (−0.03 to −0.01)	<.001	0.43
Left	Inferior occipital area (white matter)	0.30 (0.07)	0.32 (0.05)	−0.03 (−0.04 to −0.01)	<.001	0.46
Left	Inferior parietal area (white matter)	0.31 (0.07)	0.33 (0.06)	−0.03 (−0.04 to −0.01)	.001	0.39
Left	Postcentral area (white matter)	0.35 (0.05)	0.36 (0.04)	−0.02 (−0.03 to −0.01)	.001	0.39
Right	Middle occipital area (white matter)	0.30 (0.04)	0.31 (0.04)	−0.02 (−0.02 to −0.01)	.002	0.42
Left	Putamen (gray matter)	0.22 (0.03)	0.21 (0.02)	0.01 (0.005 to 0.02)	.001	0.39

Abbreviations: FA, fractional anisotropy; PAE, prenatal alcohol exposure.

^a^ No brain areas showed significant differences in other metrics (mean diffusivity, axial diffusivity, radial diffusivity, and blood oxygenation level–dependent signal temporal variance) after false discovery rate correction.

When analyses controlled for total intracranial volume, 20 brain areas, including those with differences found to be significant above, had group differences in FA. Nineteen subcortical white matter regions had significantly lower FA in the PAE group, while the putamen had higher FA in the PAE group (eTable 2 and eFigure in the Supplement).

### Associations Between CBCL Scores and MRI Metrics

**Figure 2.**
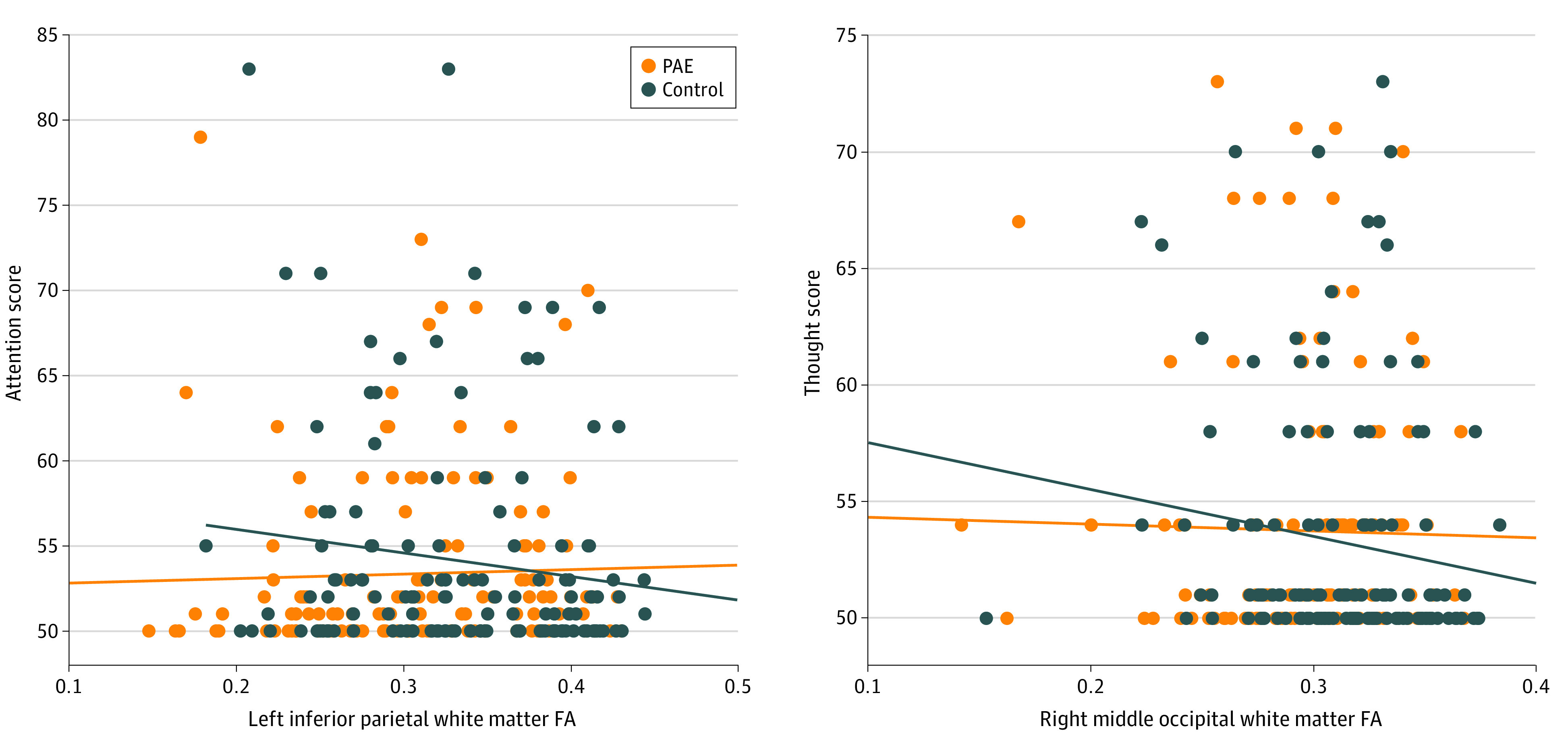
White Matter Areas With Significant Group Differences in Correlations Between Fractional Anisotropy (FA) and Child Behavior Checklist (CBCL) Scores Scatter plots for 2 selected white matter regions show different associations between FA and CBCL scores in the control group compared with the prenatal alcohol exposed (PAE) group. Trend lines are shown for both the control group (blue) and the PAE group (orange). Additional details are found in Table 3.

Among the brain regions with significant group differences on MRI metrics, FA in 2 white matter areas had significant group-behavior interactions uncorrected at *P* < .05 (Figure 2 and Table 3). In these regions, controls showed negative correlations (ρ range, −0.24 to −0.08) between FA and behavior, whereas the PAE group had no significant (ρ range, 0.02-0.16) brain-behavior associations. However, none of these findings survived multiple comparison corrections.

**Table 3.** White Matter Areas With Significant Group Differences in Correlations Between FA and CBCL Scores Uncorrected at *P* < .05

CBCL behavioral item	White matter area	PAE group		Matched unexposed controls		Mean difference in correlation coefficient (95% CI)	*P* value for mean difference	Effect size (diff rho)
		Correlation, ρ	*P* value	Correlation, ρ	*P* value			
Withdrawn/depressed	Left inferior occipital area	0.02	.79	−0.24	.004	0.27 (0.23-0.29)	.03	0.27
Rule break	Left inferior occipital area	0.16	.06	−0.08	.35	0.24 (0.23-0.24)	.05	0.24
Attention	Left inferior parietal area	0.14	.11	−0.11	.22	0.25 (0.24-0.25)	.05	0.25
Thought	Right middle occipital area	0.08	.35	−0.17	.04	0.26 (0.24-0.27)	.04	0.26
Withdrawn/depressed	Right middle occipital area	0.06	.47	−0.23	.009	0.29 (0.27-0.29)	.02	0.29

Abbreviations: CBCL, Child Behavior Checklist; FA, fractional anisotropy; PAE, prenatal alcohol exposure.

## Discussion

To our knowledge, this cross-sectional study is the first to show that small amounts (mean, 1 drink/wk) of PAE are associated with structural brain alterations in children. We found lower FA in white matter compared with unexposed control children matched for age, sex, family income, maternal educational level, caregiver status, and other prenatal substance exposures. Importantly, this finding helps provide clearer evidence of brain alterations likely resulting from PAE compared with previous studies^34,37,38,39,40^ that have sociodemographic mismatches between exposed and control groups. We also found more externalizing behavior problems in children with PAE. This is the first convincing evidence that lower levels of PAE are associated with changes in white matter and a potentially long-lasting impact on child behavior.

### Brain Alterations in Children With PAE

Our findings of lower FA are consistent with a growing literature of studies investigating children with PAE,^11,53^ although our sample had considerably lower levels of PAE than participants in prior studies. Previous studies^33,35,54^ have reported mean exposure levels of 4 to 16 drinks/wk and/or multiple binge episodes (≥4 drinks in 1 sitting), whereas our sample consumed a mean of 1 drink/wk, and most participants’ biological mothers (98.3%) reported no binge episodes during pregnancy. In fact, the mean maximum number of drinks in 1 sitting was only 1.

The subadjacent white matter of the left postcentral, parietal, and temporal regions and bilateral occipital areas showed lower FA in the PAE group compared with controls, largely in agreement with prior studies^10,11,55,56^ of children and youths with higher levels of PAE. For example, many previous studies report lower FA within the corpus callosum^10,11,55^ and left temporal lobe^56^ in children and youths with PAE. Interestingly, we observed more group differences in the left hemisphere than the right hemisphere in the present study. Cortical lateralization in white matter has not been widely studied yet in the PAE population, although a previous review found atypical asymmetry in the left temporal-parietal region associated with PAE.^57^

One prior study^29^ examined PAE in the full ABCD cohort and reported larger total brain volume and regional cortical volumes in the PAE group compared with unexposed controls. That study included participants who had been exposed to alcohol before awareness of pregnancy only, those with PAE reported by nonbiological parents, and children with other prenatal exposures (eg, cannabis, tobacco). Our study focused on a smaller but better characterized subsample of the ABCD study cohort, including only children with biological parental reports of alcohol consumption after recognition of pregnancy. Regardless, our cortical volume results are consistent, showing higher volumes in the PAE group, although we report no differences in intracranial volume in our well-matched samples. Prior studies of children with PAE consistently report lower brain volumes.^10,58^ The discrepancies may be due to different levels of PAE; prior studies have generally focused on individuals with much higher PAE than the present study. Other factors such as early-life stress, which is higher in most prior studies of PAE than in the present study, may also affect brain volumes.^29^ It is important to note that the higher cortical volume observed herein does not appear to be adaptive, because these children also had higher externalizing scores, indicating more problematic behavior.

### Associations With Behavior

The PAE group had significantly higher externalizing but not internalizing behavior scores compared with the unexposed controls. Previous studies of children, adolescents, and adults with PAE^4,23,28,29,59,60^ consistently report both more externalizing and internalizing problems, including the prior study of the full ABCD cohort.^29^ The lack of internalizing differences found herein may suggest that externalizing behaviors are more affected by smaller amounts of PAE or may be due to the fact that internalizing behaviors, because they are directed inward, can be difficult to accurately capture via parent report, especially in older children.^61,62,63^

In addition to white matter alterations in the brain, the PAE group had disrupted associations between brain structure and behavior and did not show the negative correlations found in controls. Previous studies generally show that lower FA is associated with more problematic behavior (higher internalizing and/or externalizing behavior scores) in typically developing children,^24,64^ consistent with our results herein. Prior studies of PAE^28,56^ have reported mixed findings with regard to brain-behavior associations. Sowell et al^56^ found the FA in the lateral splenium of the corpus callosum was positively correlated with visuomotor integration, whereas another study^28^ found no significant correlations between diffusion tensor imaging measures and internalizing or externalizing behavior in the group with PAE. Together, these results suggest that brain alterations induced by PAE disrupt the typical brain-behavior associations and ultimately may contribute to the behavioral difficulties widely observed in individuals with PAE.

### Structural vs Functional Alterations

No significant differences in the regional temporal variance of the functional MRI signals were found, although previous studies^13,17,19,29^ report widespread changes in functional connectivity between brain regions or networks in children with PAE. Temporal variance measures regional brain activity and thus may be less affected than connectivity between regions^65^; discrepancies between results may also reflect the lower levels of PAE reported herein compared with prior studies. More studies with combined functional and structural brain measures in individuals with PAE population will help clarify these associations.

### Implications for Policy and Research

Participants in the present study had low levels of PAE and were not clinically diagnosed with FASD. Indeed, most participants would not meet the exposure criteria for an FASD diagnosis (ie, ≥7 drinks/wk or ≥2 binge episodes during pregnancy).^66^ This highlights the importance of considering any amount of PAE, both in clinical practice and in research studies. From a policy perspective, our findings support the recommendations of the Centers for Disease Control and Prevention and the Society of Obstetricians and Gynaecologists of Canada,^67^ both of which recommend against any amount of alcohol consumption during pregnancy. Despite these recommendations, many individuals assume that small amounts of PAE are fine, and an estimated 10% of women in North America consume alcohol during pregnancy. Our results suggest that PAE status should be considered in future studies of brain alterations and behavior in any number of developmental disorders, diseases, or brain injuries, because PAE may have a substantial confounding effect on results.

### Limitations

This study has some limitations. The PAE status was obtained from a retrospective parent-report questionnaire. Retrospective self-reports of PAE can be biased and sometimes underreport alcohol consumption.^68^ To mitigate this, we limited our analysis to participants whose biological mothers reported drinking after awareness of pregnancy and compared them with participants whose biological mothers reported no alcohol consumption before or after pregnancy awareness. Another limitation was that the MRI measures were collected across multiple sites and 3 scanner types. Although the imaging protocol of the ABCD study is harmonized across scanners,^45^ MRI metrics can still vary; to mitigate this, we used a nested design in our statistical analysis.^52^

## Conclusions

In this cross-sectional study, we found structural brain alterations and worse behavior problems in children with low levels of PAE compared with a well-matched group of unexposed controls. Specifically, children with PAE had alterations in diffusion imaging metrics in left postcentral, parietal, temporal, and bilateral occipital white matter and in the gray matter of the putamen. These results suggest that even small amounts of prenatal exposure may lead to structural alterations of the brain, underscoring the importance of evidence-based policy recommendations and considering prenatal exposures in future studies of pediatric brain development.

## Notes

Supplement. **eTable 1.** Group Comparisons on the CBCL Measurements **eTable 2.** Brain Areas With Significant FA Differences Between Groups After FDR Correction When Controlling for Total Intracranial Volume **eFigure.** Brain Areas With Significant FA Differences Between Groups After FDR Correction When Controlling for Total Intracranial Volume

## References

[1] Popova S, Lange S, Probst C, Gmel G, Rehm J. Estimation of national, regional, and global prevalence of alcohol use during pregnancy and fetal alcohol syndrome: a systematic review and meta-analysis. Lancet Glob Health. 2017;5(3):e290-e299. doi:10.1016/S2214-109X(17)30021-9 28089487

[2] Riley EP, Infante MA, Warren KR. Fetal alcohol spectrum disorders: an overview. Neuropsychol Rev. 2011;21(2):73-80. doi:10.1007/s11065-011-9166-x 21499711 PMC3779274

[3] Lynch ME, Kable JA, Coles CD. Effects of prenatal alcohol exposure in a prospective sample of young adults: mental health, substance use, and difficulties with the legal system. Neurotoxicol Teratol. 2017;64:50-62. doi:10.1016/j.ntt.2017.10.001 28986209 PMC5739524

[4] Pei J, Denys K, Hughes J, Rasmussen C. Mental health issues in fetal alcohol spectrum disorder. J Ment Health. 2011;20(5):438-448. doi:10.3109/09638237.2011.577113 21780939

[5] Lebel C, Roussotte F, Sowell ER. Imaging the impact of prenatal alcohol exposure on the structure of the developing human brain. Neuropsychol Rev. 2011;21(2):102-118. doi:10.1007/s11065-011-9163-0 21369875 PMC3098972

[6] Donald KA, Fouche JP, Roos A, . Alcohol exposure in utero is associated with decreased gray matter volume in neonates. Metab Brain Dis. 2016;31(1):81-91. doi:10.1007/s11011-015-9771-0 26616173 PMC6556617

[7] Donald KA, Roos A, Fouche JP, . A study of the effects of prenatal alcohol exposure on white matter microstructural integrity at birth. Acta Neuropsychiatr. 2015;27(4):197-205. doi:10.1017/neu.2015.35 26022619 PMC6465963

[8] Kar P, Reynolds JE, Grohs MN, . White matter alterations in young children with prenatal alcohol exposure. Dev Neurobiol. 2021;81(4):400-410. doi:10.1002/dneu.22821 33829663

[9] Moore EM, Migliorini R, Infante MA, Riley EP. Fetal alcohol spectrum disorders: recent neuroimaging findings. Curr Dev Disord Rep. 2014;1(3):161-172. doi:10.1007/s40474-014-0020-8 25346882 PMC4207054

[10] Nguyen VT, Chong S, Tieng QM, Mardon K, Galloway GJ, Kurniawan ND. Radiological studies of fetal alcohol spectrum disorders in humans and animal models: an updated comprehensive review. Magn Reson Imaging. 2017;43:10-26. doi:10.1016/j.mri.2017.06.012 28645698

[11] Wozniak JR, Muetzel RL. What does diffusion tensor imaging reveal about the brain and cognition in fetal alcohol spectrum disorders? Neuropsychol Rev. 2011;21(2):133-147. doi:10.1007/s11065-011-9162-1 21347880

[12] Long X, Little G, Treit S, Beaulieu C, Gong G, Lebel C. Altered brain white matter connectome in children and adolescents with prenatal alcohol exposure. Brain Struct Funct. 2020;225(3):1123-1133. doi:10.1007/s00429-020-02064-z 32239277

[13] Donald KA, Ipser JC, Howells FM, . Interhemispheric functional brain connectivity in neonates with prenatal alcohol exposure: preliminary findings. Alcohol Clin Exp Res. 2016;40(1):113-121. doi:10.1111/acer.12930 26727529 PMC6556616

[14] Roos A, Fouche JP, Ipser JC, . Structural and functional brain network alterations in prenatal alcohol exposed neonates. Brain Imaging Behav. 2021;15(2):689-699. doi:10.1007/s11682-020-00277-8 32306280 PMC7572489

[15] Santhanam P, Coles CD, Li Z, Li L, Lynch ME, Hu X. Default mode network dysfunction in adults with prenatal alcohol exposure. Psychiatry Res. 2011;194(3):354-362. doi:10.1016/j.pscychresns.2011.05.004 22079659 PMC3225604

[16] Long X, Little G, Beaulieu C, Lebel C. Sensorimotor network alterations in children and youth with prenatal alcohol exposure. Hum Brain Mapp. 2018;39(5):2258-2268. doi:10.1002/hbm.24004 29436054 PMC6866525

[17] Fan J, Taylor PA, Jacobson SW, . Localized reductions in resting-state functional connectivity in children with prenatal alcohol exposure. Hum Brain Mapp. 2017;38(10):5217-5233. doi:10.1002/hbm.23726 28734059 PMC6377933

[18] Infante MA, Moore EM, Bischoff-Grethe A, Tapert SF, Mattson SN, Riley EP. Altered functional connectivity during spatial working memory in children with heavy prenatal alcohol exposure. Alcohol. 2017;64:11-21. doi:10.1016/j.alcohol.2017.05.002 28965651 PMC5635832

[19] Little G, Reynolds J, Beaulieu C. Altered functional connectivity observed at rest in children and adolescents prenatally exposed to alcohol. Brain Connect. 2018;8(8):503-515. doi:10.1089/brain.2017.0572 30289280

[20] Ware AL, Long X, Lebel C. Functional connectivity of the attention networks is altered and relates to neuropsychological outcomes in children with prenatal alcohol exposure. Dev Cogn Neurosci. 2021;48:100951. doi:10.1016/j.dcn.2021.100951 33838597 PMC8044997

[21] Wozniak JR, Mueller BA, Bell CJ, . Global functional connectivity abnormalities in children with fetal alcohol spectrum disorders. Alcohol Clin Exp Res. 2013;37(5):748-756. doi:10.1111/acer.12024 23240997 PMC3610852

[22] Wozniak JR, Mueller BA, Mattson SN, ; CIFASD. Functional connectivity abnormalities and associated cognitive deficits in fetal alcohol spectrum disorders (FASD). Brain Imaging Behav. 2017;11(5):1432-1445. doi:10.1007/s11682-016-9624-4 27734306 PMC5389933

[23] Khoury JE, Jamieson B, Milligan K. Risk for childhood internalizing and externalizing behavior problems in the context of prenatal alcohol exposure: a meta-analysis and comprehensive examination of moderators. Alcohol Clin Exp Res. 2018;42(8):1358-1377. doi:10.1111/acer.13805 29852057

[24] Andre QR, Geeraert BL, Lebel C. Brain structure and internalizing and externalizing behavior in typically developing children and adolescents. Brain Struct Funct. 2020;225(4):1369-1378. doi:10.1007/s00429-019-01973-y 31701264

[25] Whittle S, Vijayakumar N, Simmons JG, Allen NB. Internalizing and externalizing symptoms are associated with different trajectories of cortical development during late childhood. J Am Acad Child Adolesc Psychiatry. 2020;59(1):177-185. doi:10.1016/j.jaac.2019.04.006 31047992

[26] Koolschijn PCMP, van IJzendoorn MH, Bakermans-Kranenburg MJ, Crone EA. Hippocampal volume and internalizing behavior problems in adolescence. Eur Neuropsychopharmacol. 2013;23(7):622-628. doi:10.1016/j.euroneuro.2012.07.001 22824414

[27] Snyder HR, Hankin BL, Sandman CA, Head K, Davis EP. Distinct patterns of reduced prefrontal and limbic grey matter volume in childhood general and internalizing psychopathology. Clin Psychol Sci. 2017;5(6):1001-1013. doi:10.1177/2167702617714563 29399423 PMC5794221

[28] Andre QR, McMorris CA, Kar P, . Different brain profiles in children with prenatal alcohol exposure with or without early adverse exposures. Hum Brain Mapp. 2020;41(15):4375-4385. doi:10.1002/hbm.25130 32659051 PMC7502833

[29] Lees B, Mewton L, Jacobus J, . Association of prenatal alcohol exposure with psychological, behavioral, and neurodevelopmental outcomes in children from the Adolescent Brain Cognitive Development study. Am J Psychiatry. 2020;177(11):1060-1072. doi:10.1176/appi.ajp.2020.20010086 32972200 PMC7924902

[30] Comasco E, Rangmar J, Eriksson UJ, Oreland L. Neurological and neuropsychological effects of low and moderate prenatal alcohol exposure. Acta Physiol (Oxf). 2018;222(1):e12892. doi:10.1111/apha.12892 28470828

[31] Mukherjee RAS, Hollins S, Abou-Saleh MT, Turk J. Low level alcohol consumption and the fetus. BMJ. 2005;330(7488):375-376. doi:10.1136/bmj.330.7488.375 15718517 PMC549096

[32] Rodriguez CI, Davies S, Calhoun V, Savage DD, Hamilton DA. Moderate prenatal alcohol exposure alters functional connectivity in the adult rat brain. Alcohol Clin Exp Res. 2016;40(10):2134-2146. doi:10.1111/acer.13175 27570053 PMC5048527

[33] Lebel C, Mattson SN, Riley EP, . A longitudinal study of the long-term consequences of drinking during pregnancy: heavy in utero alcohol exposure disrupts the normal processes of brain development. J Neurosci. 2012;32(44):15 243-15 251. doi:10.1523/JNEUROSCI.1161-12.2012 23115162 PMC3515671

[34] Astley SJ, Aylward EH, Olson HC, . Magnetic resonance imaging outcomes from a comprehensive magnetic resonance study of children with fetal alcohol spectrum disorders. Alcohol Clin Exp Res. 2009;33(10):1671-1689. doi:10.1111/j.1530-0277.2009.01004.x 19572986 PMC4170878

[35] Chen X, Coles CD, Lynch ME, Hu X. Understanding specific effects of prenatal alcohol exposure on brain structure in young adults. Hum Brain Mapp. 2012;33(7):1663-1676. doi:10.1002/hbm.21313 21692145 PMC3755753

[36] Archibald SL, Fennema-Notestine C, Gamst A, Riley EP, Mattson SN, Jernigan TL. Brain dysmorphology in individuals with severe prenatal alcohol exposure. Dev Med Child Neurol. 2001;43(3):148-154. doi:10.1111/j.1469-8749.2001.tb00179.x 11263683

[37] McLachlan K, Zhou D, Little G, . Current socioeconomic status correlates with brain volumes in healthy children and adolescents but not in children with prenatal alcohol exposure. Front Hum Neurosci. 2020;14:223. doi:10.3389/fnhum.2020.00223 32714166 PMC7344164

[38] Krueger AM, Roediger DJ, Mueller BA, . Para-limbic structural abnormalities are associated with internalizing symptoms in children with prenatal alcohol exposure. Alcohol Clin Exp Res. 2020;44(8):1598-1608. doi:10.1111/acer.14390 32524616 PMC7484415

[39] Whaley SE, O’Connor MJ. Increasing the report of alcohol use among low-income pregnant women. Am J Health Promot. 2003;17(6):369-372. doi:10.4278/0890-1171-17.6.369 12858616

[40] Robinson M, Oddy WH, McLean NJ, . Low-moderate prenatal alcohol exposure and risk to child behavioural development: a prospective cohort study. BJOG. 2010;117(9):1139-1150. doi:10.1111/j.1471-0528.2010.02596.x 20528867

[41] Popova S, Temple V, Dozet D, O’Hanlon G, Toews C, Rehm J. Health, social and legal outcomes of individuals with diagnosed or at risk for fetal alcohol spectrum disorder: Canadian example. Drug Alcohol Depend. 2021;219:108487. doi:10.1016/j.drugalcdep.2020.108487 33385689

[42] Scott-Goodwin AC, Puerto M, Moreno I. Toxic effects of prenatal exposure to alcohol, tobacco and other drugs. Reprod Toxicol. 2016;61:120-130. doi:10.1016/j.reprotox.2016.03.043 27037188

[43] Irner TB. Substance exposure in utero and developmental consequences in adolescence: a systematic review. Child Neuropsychol. 2012;18(6):521-549. doi:10.1080/09297049.2011.628309 22114955

[44] Williams JHG, Ross L. Consequences of prenatal toxin exposure for mental health in children and adolescents: a systematic review. Eur Child Adolesc Psychiatry. 2007;16(4):243-253. doi:10.1007/s00787-006-0596-6 17200791

[45] Casey BJ, Cannonier T, Conley MI, ; ABCD Imaging Acquisition Workgroup. The Adolescent Brain Cognitive Development (ABCD) study: imaging acquisition across 21 sites. Dev Cogn Neurosci. 2018;32:43-54. doi:10.1016/j.dcn.2018.03.001 29567376 PMC5999559

[46] Auchter AM, Hernandez Mejia M, Heyser CJ, . A description of the ABCD organizational structure and communication framework. Dev Cogn Neurosci. 2018;32:8-15. doi:10.1016/j.dcn.2018.04.003 29706313 PMC6462277

[47] Barch DM, Albaugh MD, Avenevoli S, . Demographic, physical and mental health assessments in the Adolescent Brain and Cognitive Development study: rationale and description. Dev Cogn Neurosci. 2018;32:55-66. doi:10.1016/j.dcn.2017.10.010 29113758 PMC5934320

[48] Hagler DJ Jr, Hatton S, Cornejo MD, . Image processing and analysis methods for the Adolescent Brain Cognitive Development study. Neuroimage. 2019;202:116091. doi:10.1016/j.neuroimage.2019.116091 31415884 PMC6981278

[49] Fischl B, Salat DH, Busa E, . Whole brain segmentation: automated labeling of neuroanatomical structures in the human brain. Neuron. 2002;33(3):341-355. doi:10.1016/S0896-6273(02)00569-X 11832223

[50] Elman JA, Panizzon MS, Hagler DJ Jr, . Genetic and environmental influences on cortical mean diffusivity. Neuroimage. 2017;146:90-99. doi:10.1016/j.neuroimage.2016.11.032 27864081 PMC5322245

[51] Destrieux C, Fischl B, Dale A, Halgren E. Automatic parcellation of human cortical gyri and sulci using standard anatomical nomenclature. Neuroimage. 2010;53(1):1-15. doi:10.1016/j.neuroimage.2010.06.010 20547229 PMC2937159

[52] Heeringa SG, Berglund PA. A guide for population-based analysis of the Adolescent Brain Cognitive Development (ABCD) study baseline data. bioRxiv. Preprint posted online February 10, 2020. doi:10.1101/2020.02.10.942011

[53] Lebel C, Rasmussen C, Wyper K, . Brain diffusion abnormalities in children with fetal alcohol spectrum disorder. Alcohol Clin Exp Res. 2008;32(10):1732-1740. doi:10.1111/j.1530-0277.2008.00750.x 18671811

[54] De Guio F, Mangin JF, Rivière D, . A study of cortical morphology in children with fetal alcohol spectrum disorders. Hum Brain Mapp. 2014;35(5):2285-2296. doi:10.1002/hbm.22327 23946151 PMC6869611

[55] Wozniak JR, Muetzel RL, Mueller BA, . Microstructural corpus callosum anomalies in children with prenatal alcohol exposure: an extension of previous diffusion tensor imaging findings. Alcohol Clin Exp Res. 2009;33(10):1825-1835. doi:10.1111/j.1530-0277.2009.01021.x 19645729 PMC2895908

[56] Sowell ER, Johnson A, Kan E, . Mapping white matter integrity and neurobehavioral correlates in children with fetal alcohol spectrum disorders. J Neurosci. 2008;28(6):1313-1319. doi:10.1523/JNEUROSCI.5067-07.2008 18256251 PMC3567846

[57] Lindell AK. Atypical hemispheric asymmetry in fetal alcohol spectrum disorders: a review of the effects of prenatal alcohol exposure on language lateralization. Acta Neuropsychol. 2016;14(4):367-380. doi:10.5604/17307503.1227531

[58] Donald KA, Eastman E, Howells FM, . Neuroimaging effects of prenatal alcohol exposure on the developing human brain: a magnetic resonance imaging review. Acta Neuropsychiatr. 2015;27(5):251-269. doi:10.1017/neu.2015.12 25780875

[59] Day NL, Helsel A, Sonon K, Goldschmidt L. The association between prenatal alcohol exposure and behavior at 22 years of age. Alcohol Clin Exp Res. 2013;37(7):1171-1178. doi:10.1111/acer.12073 23442183

[60] Gautam P, Lebel C, Narr KL, . Volume changes and brain-behavior relationships in white matter and subcortical gray matter in children with prenatal alcohol exposure. Hum Brain Mapp. 2015;36(6):2318-2329. doi:10.1002/hbm.22772 25711175 PMC4631525

[61] Duhig AM, Renk K, Epstein MK, Phares V. Interparental agreement on internalizing, externalizing, and total behavior problems: a meta-analysis. Clin Psychol Sci Pract. 2000;7(4):435-453. doi:10.1093/clipsy.7.4.435

[62] De Los Reyes A, Augenstein TM, Wang M, . The validity of the multi-informant approach to assessing child and adolescent mental health. Psychol Bull. 2015;141(4):858-900. doi:10.1037/a0038498 25915035 PMC4486608

[63] Silverman WK, Eisen AR. Age differences in the reliability of parent and child reports of child anxious symptomatology using a structured interview. J Am Acad Child Adolesc Psychiatry. 1992;31(1):117-124. doi:10.1097/00004583-199201000-00018 1537762

[64] Mohamed Ali O, Vandermeer MRJ, Sheikh HI, Joanisse MF, Hayden EP. Girls’ internalizing symptoms and white matter tracts in cortico-limbic circuitry. Neuroimage Clin. 2019;21:101650. doi:10.1016/j.nicl.2018.101650 30611742 PMC6412069

[65] Duff EP, Makin T, Cottaar M, Smith SM, Woolrich MW. Disambiguating brain functional connectivity. Neuroimage. 2018;173(January):540-550. doi:10.1016/j.neuroimage.2018.01.053 29476911 PMC5929905

[66] Chudley AE, Conry J, Cook JL, Loock C, Rosales T, LeBlanc N; Public Health Agency of Canada’s National Advisory Committee on Fetal Alcohol Spectrum Disorder. Fetal alcohol spectrum disorder: Canadian guidelines for diagnosis. CMAJ. 2005;172(5)(suppl):S1-S21. doi:10.1503/cmaj.1040302 15738468 PMC557121

[67] Carson G, Cox LV, Crane J, ; Society of Obstetricians and Gynaecologists of Canada. Alcohol use and pregnancy consensus clinical guidelines. J Obstet Gynaecol Can. 2010;32(8)(suppl 3):S1-S31. doi:10.1016/S1701-2163(16)34633-3 21172102

[68] Lange S, Shield K, Koren G, Rehm J, Popova S. A comparison of the prevalence of prenatal alcohol exposure obtained via maternal self-reports versus meconium testing: a systematic literature review and meta-analysis. BMC Pregnancy Childbirth. 2014;14(1):127. doi:10.1186/1471-2393-14-127 24708684 PMC3992148

