# Supplementary material for: Evaluation of Brain Alterations and Behavior in Children With Low Levels of Prenatal Alcohol Exposure
Source: JAMA Netw Open. 2022 Apr 5;5(4):e225972. doi: 10.1001/jamanetworkopen.2022.5972 (PMC8984786; doi:10.1001/jamanetworkopen.2022.5972)
Supplement: Supplement. — eTable 1. Group Comparisons on the CBCL Measurements eTable 2. Brain Areas With Significant FA Differences Between Groups After FDR Correction When Controlling for Total Intracranial Volume eFigure. Brain Areas With Significant FA Differences Between Groups After FDR Correction When Controlling for Total Intracranial Volume [file jamanetwopen-e225972-s001.pdf]

Supplementary Online Content

Long X, Lebel C. Evaluation of brain alterations and behavior in children with low levels of prenatal alcohol exposure. *JAMA Netw Open*. 2022;5(4):e225972. doi:10.1001/jamanetworkopen.2022.5972

eTable 1. Group Comparisons on the CBCL Measurements
eTable 2. Brain Areas With Significant FA Differences Between Groups After FDR Correction When Controlling For Total Intracranial Volume
eFigure. Brain Areas With Significant FA Differences Between Groups After FDR Correction When Controlling For Total Intracranial Volume

This supplementary material has been provided by the authors to give readers additional information about their work.

© 2022 Long X et al. *JAMA Network Open*.

eTable 1. Group Comparisons on the CBCL Measurements

The CBCL scores are t-scores. Bold values indicate the results at $p < 0.05$, uncorrected.

CBCL items	PAE	Matched UC	Mean differences with 95% CI	p-value	Cohen's d	PAE% (>= 65)	Matched UC% (>= 65)
**Internalizing**	49.02±9.97	48.4±10.35	0.62 [-1.81 3.06]	0.62	0.06	6.67	7.41
**Externalizing**	**45.19±8.75**	**42.81±8.63**	**2.39 [0.30 4.47]**	**0.03**	**0.27**	**1.48**	**0.00**
**Anxiety depression**	53.40±6.22	53.56±5.74	-0.16 [-1.59 1.28]	0.83	0.03	5.93	7.41
**Withdraw depression**	53.11±5.21	53.03±5.65	0.08 [-1.22 1.38]	0.90	0.01	6.67	5.93
**Somatic**	55.47±6.17	54.98±5.64	0.49 [-0.93 1.91]	0.50	0.08	8.15	6.67
**Social**	51.71±3.61	52.53±4.56	-0.82 [-1.81 0.16]	0.10	0.20	2.96	2.96
**Thought**	53.80±5.43	53.27±5.30	0.53 [-0.76 1.81]	0.42	0.10	6.67	6.67
**Attention**	53.47±5.41	54.11±6.52	-0.64 [-2.08 0.79]	0.38	0.11	5.19	9.63
**Rule Break**	51.88±3.60	51.36±3.24	0.53 [-0.30 1.35]	0.21	0.15	2.22	1.48
**Aggressive**	52.10±3.99	51.58±3.30	0.53 [-0.35 1.40]	0.24	0.14	1.48	0.74
**Total problem**	45.77±9.40	44.64±10.46	1.13 [-1.26 3.51]	0.35	0.11	3.70	3.70

© 2022 Long X et al. *JAMA Network Open*.

eTable 2. Brain Areas With Significant FA Differences Between Groups After FDR Correction When Controlling For Total Intracranial Volume

Bold rows indicate the results that were also significant when total intracranial volume was not controlled (i.e., results in main manuscript).

Hemisphere	Brain region (white matter)	PAE FA	Control FA	Mean differences with 95% CI	p-value	Cohen's d
Left	Paracentral lobule	0.25±0.04	0.27±0.03	-0.01 [-0.02 -0.004]	0.004	0.33
Left	Middle cingulate area	0.37±0.05	0.39±0.04	-0.02 [-0.03 -0.005]	0.006	0.34
Left	Middle frontal area	0.23±0.04	0.24±0.03	-0.01 [-0.02 -0.004]	0.003	0.33
Left	Superior frontal	0.27±0.03	0.28±0.03	-0.01 [-0.02 -0.004]	0.004	0.33
Left	Middle occipital area	0.19±0.03	0.21±0.03	-0.01 [-0.02 -0.004]	0.001	0.44
Left	Angular area	0.22±0.03	0.23±0.03	-0.01 [-0.02 -0.005]	0.001	0.43
Left	Supramarginal area	0.23±0.03	0.24±0.03	-0.01 [-0.02 -0.004]	0.003	0.40
Left	Postcentral gyrus area	0.22±0.03	0.23±0.03	-0.01 [-0.02 -0.004]	0.003	0.33
Left	Precentral area	0.26±0.04	0.27±0.04	-0.01 [-0.02 -0.01]	0.002	0.36
**Left**	**Planum temporale**	**0.26±0.04**	**0.28±0.03**	**-0.02 [-0.03 -0.01]**	**<0.001**	**0.43**
Left	Anterior lateral sulcus area	0.30±0.07	0.32±0.05	-0.02 [-0.03 -0.01]	0.005	0.37
Left	Central sulcus area	0.38±0.05	0.39±0.04	-0.02 [-0.03 -0.01]	0.002	0.37
**Left**	**Inferior occipital area**	**0.30±0.07**	**0.32±0.05**	**-0.03 [-0.04 -0.01]**	**<0.001**	**0.46**
**Left**	**Inferior parietal area**	**0.31±0.07**	**0.33±0.06**	**-0.03 [-0.04 -0.01]**	**0.001**	**0.39**
Left	Superior occipital area	0.37±0.05	0.39±0.04	-0.02 [-0.03 -0.01]	0.003	0.34
**Left**	**Postcentral area**	**0.35±0.05**	**0.36±0.04**	**-0.02 [-0.03 -0.01]**	**0.001**	**0.39**
Left	Superior precentral area	0.40±0.05	0.41±0.04	-0.01 [-0.03 -0.005]	0.004	0.33

© 2022 Long X et al. *JAMA Network Open*.

Right	Superior parietal lobule	0.23±0.04	0.24±0.03	-0.01 [-0.02 -0.01]	0.001	0.36
**Right**	**Middle occipital area**	**0.30±0.04**	**0.31±0.04**	**-0.01 [-0.02 -0.01]**	**0.001**	**0.39**
**Left**	**Putamen (grey matter)**	**0.22±0.03**	**0.21±0.02**	**0.01 [0.005 0.02]**	**0.001**	**0.42**

© 2022 Long X et al. *JAMA Network Open*.

eFigure. Brain Areas With Significant FA Differences Between Groups After FDR Correction When Controlling For Total Intracranial Volume

White matter areas with lower FA in the PAE group compared to unexposed controls are shown in blue.

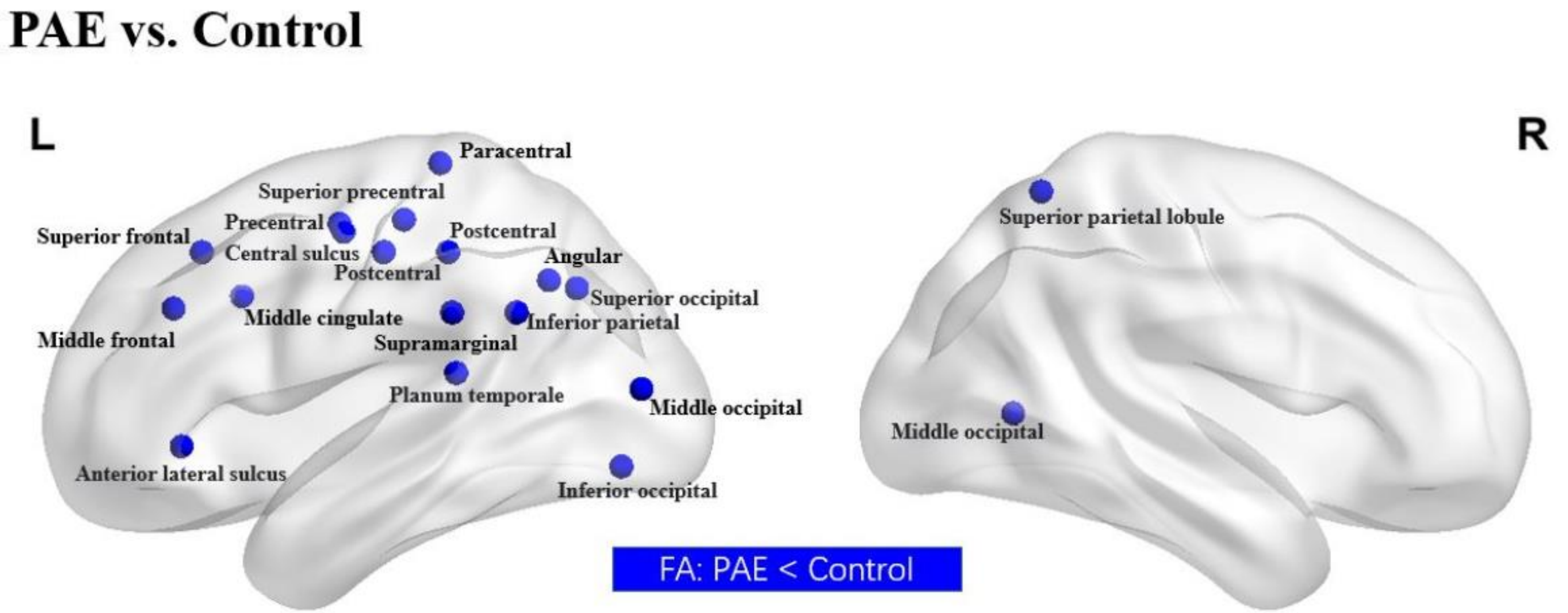

© 2022 Long X et al. *JAMA Network Open*.
